# On the equivalence between marker effect models and breeding value models and direct genomic values with the Algorithm for Proven and Young

**DOI:** 10.1186/s12711-022-00741-7

**Published:** 2022-07-16

**Authors:** Matias Bermann, Daniela Lourenco, Natalia S. Forneris, Andres Legarra, Ignacy Misztal

**Affiliations:** 1 Department of Animal and Dairy Science, University of Georgia, Athens, GA 30602 USA; 2 Facultad de Agronomía, Universidad de Buenos Aires, C1417DSQ Buenos Aires, Argentina; 3 Instituto de Investigaciones en Producción Animal (INPA), CONICET - Universidad de Buenos Aires, C1427CWO Buenos Aires, Argentina; 4 INRA, UMR1388 GenPhySE, 31326 Castanet‐Tolosan, France

## Abstract

**Background:**

Single-step genomic predictions obtained from a breeding value model require calculating the inverse of the genomic relationship matrix $({\mathbf{G}}^{-1})$. The Algorithm for Proven and Young (APY) creates a sparse representation of ${\mathbf{G}}^{-1}$ with a low computational cost. APY consists of selecting a group of core animals and expressing the breeding values of the remaining animals as a linear combination of those from the core animals plus an error term. The objectives of this study were to: (1) extend APY to marker effects models; (2) derive equations for marker effect estimates when APY is used for breeding value models, and (3) show the implication of selecting a specific group of core animals in terms of a marker effects model.

**Results:**

We derived a family of marker effects models called APY-SNP-BLUP. It differs from the classic marker effects model in that the row space of the genotype matrix is reduced and an error term is fitted for non-core animals. We derived formulas for marker effect estimates that take this error term in account. The prediction error variance (PEV) of the marker effect estimates depends on the PEV for core animals but not directly on the PEV of the non-core animals. We extended the APY-SNP-BLUP to include a residual polygenic effect and accommodate non-genotyped animals. We show that selecting a specific group of core animals is equivalent to select a subspace of the row space of the genotype matrix. As the number of core animals increases, subspaces corresponding to different sets of core animals tend to overlap, showing that random selection of core animals is algebraically justified.

**Conclusions:**

The APY-(ss)GBLUP models can be expressed in terms of marker effect models. When the number of core animals is equal to the rank of the genotype matrix, APY-SNP-BLUP is identical to the classic marker effects model. If the number of core animals is less than the rank of the genotype matrix, genotypes for non-core animals are imputed as a linear combination of the genotypes of the core animals. For estimating SNP effects, only relationships and estimated breeding values for core animals are needed.

**Supplementary Information:**

The online version contains supplementary material available at 10.1186/s12711-022-00741-7.

## Background

Genomic predictions can be obtained from either a breeding value model or a marker effects model [1]. The equivalence between these two models [2] facilitates interpretation in terms of either marker effects or genomic relationships between individuals. On the one hand, from a computational point of view, breeding value models fit a random effect, with the number of levels equal to the number of genotyped animals $\left({n}_{gt}\right)$. On the other hand, marker effects models fit a random effect, with the number of levels equal to the number of markers $\left(m\right)$. To obtain predictions for the first type of models using Henderson’s mixed model equations (MME), the inverse of the genomic relationship matrix $\left(\mathbf{G}\right)$ is needed [3]. However, direct inversion of $\mathbf{G}$ when ${n}_{gt}$ is large is very expensive or even not feasible—for instance, genomic evaluation data for US dairy cattle data contain millions of genotyped animals. In contrast, marker effect models do not suffer from this constraint because the size of the block of equations concerning marker effects remains constant. Besides this advantage, marker effect models require multiplications of dense matrices and the condition number (i.e. the quotient between the largest and smallest eigenvalue of the MME) of the system is larger than for the breeding value models [4]. To handle these issues, complex solvers [5] and refined convergence criteria [6] are needed.

Increasing numbers of animals are genotyped every year but the number of markers ($m$) remains constant at around 50K [7], and therefore the rank of $\mathbf{G}$ is (at most) 50K. To make the breeding value models feasible with large ${n}_{gt}$, several strategies have been proposed. Misztal et al. [8] and Misztal [9] developed the Algorithm for Proven and Young (APY), which uses a sparse representation of ${\mathbf{G}}^{-1}$. The APY consists of selecting a group of genotyped animals, known as *core* animals, which are selected to span roughly 99% of the eigenvalue spectra of $\mathbf{G}$, and then expressing the breeding values of the remaining genotyped animals, known as *non-core* animals, as a linear function of the breeding values of the core animals plus an error term. Mäntysaari et al. [10] proposed the GT best linear unbiased predictor (GTBLUP) model, which uses the Woodbury Identity [11] to obtain the inverse of $\mathbf{G}$ plus a regularization matrix without explicitly computing ${\mathbf{G}}^{-1}$. The GTBLUP model requires more operations than APY when the number of core animals is less than $m$ [10]. Fernando et al. [12] reviewed and developed different alternatives to the APY. In their study, the most practical implementation (Strategy IV) consisted of fitting a model with a design matrix that results from orthonormalization of the rows of the genotype matrix, and then obtaining the estimated breeding values as the product between the design matrix and the vector of solutions. APY, GTBLUP, and Strategy IV were compared in several scenarios and gave similar predictions when the spectrum of $\mathbf{G}$ was well covered by core animals [10, 13].

The lack of clear-cut, deterministic selection criteria for core animals in APY has been criticized [10, 12]; however, there are empirical studies on which [14] and how many [15] animals to include in the core group. However, the implications of using APY in terms of indirect predictions (direct genomic values) or single nucleotide polymorphism (SNP) effects are also unclear.

Although there are empirical results showing the reliable behavior of APY for prediction, an analytical framework to justify and examine its properties is lacking. Thus, the objective of this study was to derive a marker effects model that is equivalent to the APY. Departing from such a model, the secondary objectives of this study were to derive appropriate formulae for SNP and indirect predictions for genotyped animals without progeny and records, and to show the theoretical implications of selecting a specific group of core animals in APY.

## Theory

Let $$\mathbf{u}\mathbf{^{\prime}}=\left[\begin{array}{cc}{\mathbf{u}}_{\text{c}}^{\mathbf{^{\prime}}}& {\mathbf{u}}_{\text{n}}^{\mathbf{^{\prime}}}\end{array}\right]\mathbf{^{\prime}}$$ be the vector of breeding values, where ${\mathbf{u}}_{\text{c}}$ represents the sub-vector of breeding values for the core animals and ${\mathbf{u}}_{\text{n}}$ is the sub-vector of breeding values for the non-core animals. Hereafter, the sub or superscript $c$ will denote an object belonging to the core animals, whereas the sub or superscript $n$ will indicate that an object belongs to the non-core animals. For a conventional GBLUP model [1], the covariance matrix of $\mathbf{u}$, assuming a genetic variance equal to unity, is equal to:

1 $$\text{Var}\left[\begin{array}{c}{\mathbf{u}}_{\text{c}}\\ {\mathbf{u}}_{\text{n}}\end{array}\right]=\mathbf{G}=\left[\begin{array}{cc}{\mathbf{G}}_{\text{cc}}&{\mathbf{G}}_{\text{cn}}\\ {\mathbf{G}}_{\text{nc}}& {\mathbf{G}}_{\text{nn}}\end{array}\right]=\left[\begin{array}{cc}k{\mathbf{Z}}_{\text{c}}{\mathbf{Z}}_{\text{c}}^{{^{\prime}}}&k{\mathbf{Z}}_{\text{c}}{\mathbf{Z}}_{\text{n}}^{{^{\prime}}}\\ k{\mathbf{Z}}_{\text{n}}{\mathbf{Z}}_{\text{c}}^{{^{\prime}}}&k{\mathbf{Z}}_{\text{n}}{\mathbf{Z}}_{\text{n}}^{{^{\prime}}}\end{array}\right]=k\mathbf{Z}\mathbf{Z},$$

where $k$ is a scaling factor based on the level of heterozygosity (for instance: 1/$2\sum {p}_{i}{q}_{i}$) or on the number of markers, and $$\mathbf{Z}=\left[\begin{array}{c}{\mathbf{Z}}_{\text{c}}\\ {\mathbf{Z}}_{\text{n}}\end{array}\right]$$ is the matrix of genotypes for all the genotyped animals. Here it is assumed that $\mathbf{Z}$ was derived after genotype quality control [16], centering [2], and/or additional scaling [17]. Furthermore, it is assumed that the number of core animals is smaller than or equal to the number of markers and that the core animals are chosen such that ${\mathbf{G}}_{\text{cc}}$ is non-singular.

The APY approach is based on the following recursion [9]:

2 $$\left[\begin{array}{c}{\mathbf{u}}_{\text{c}}\\ {\mathbf{u}}_{\text{n}}\end{array}\right]=\left[\begin{array}{cc}\mathbf{I}& \boldsymbol{0}\\ {\mathbf{P}}_{\text{nc}}& \mathbf{I}\end{array}\right]\left[\begin{array}{c}{\mathbf{u}}_{\text{c}}\\ {\varvec{\upxi}}\end{array}\right],$$

where ${\mathbf{P}}_{\text{nc}}={\mathbf{G}}_{\text{nc}}{\mathbf{G}}_{\text{cc}}^{-1}$, and ${\varvec{\upxi}}$ is an error term that is assumed to be independent of ${\mathbf{u}}_{\text{c}}$. Taking the variance of both sides in Eq. (2), with their respective inverses, leads to:

$$\text{Var}\left[\begin{array}{c}{\mathbf{u}}_{\text{c}}\\ {\mathbf{u}}_{\text{n}}\end{array}\right]={\mathbf{G}}_{\text{APY}}=\left[\begin{array}{cc}\mathbf{I}& \boldsymbol{0}\\ {\mathbf{P}}_{\text{nc}}& \mathbf{I}\end{array}\right]\left[\begin{array}{cc}{\mathbf{G}}_{\text{cc}}& \boldsymbol{0}\\ \boldsymbol{0}& {\mathbf{M}}_{\text{nn}}\end{array}\right]\left[\begin{array}{cc}\mathbf{I}& {\mathbf{P}}_{\text{cn}}\\ \boldsymbol{0}& \mathbf{I}\end{array}\right]=\left[\begin{array}{cc}{\mathbf{G}}_{\text{cc}}& {\mathbf{G}}_{\text{cn}}\\ {\mathbf{G}}_{\text{nc}}& {\mathbf{M}}_{\text{nn}}+{{\mathbf{G}}_{\text{nc}}{\mathbf{G}}_{\text{cc}}^{-1}\mathbf{G}}_{\text{cn}}\end{array}\right],$$

3 $${\text{Var}\left[\begin{array}{c}{\mathbf{u}}_{\text{c}}\\ {\mathbf{u}}_{\text{n}}\end{array}\right]}^{-1}={\mathbf{G}}_{\text{APY}}^{-1}=\left[\begin{array}{cc}\mathbf{I}& -{\mathbf{P}}_{\text{cn}}\\ \boldsymbol{0}& \mathbf{I}\end{array}\right]\left[\begin{array}{cc}{\mathbf{G}}_{\text{cc}}^{-1}& \boldsymbol{0}\\ \boldsymbol{0}& {\mathbf{M}}_{\text{nn}}^{-1}\end{array}\right]\left[\begin{array}{cc}\mathbf{I}& \boldsymbol{0}\\ -{\mathbf{P}}_{\text{nc}}& \mathbf{I}\end{array}\right]=\left[\begin{array}{cc}{\mathbf{G}}_{\text{cc}}^{-1}+{\mathbf{P}}_{\text{cn}}{\mathbf{M}}_{\text{nn}}^{-1}{\mathbf{P}}_{\text{nc}}& -{\mathbf{P}}_{\text{cn}}{\mathbf{M}}_{\text{nn}}^{-1}\\ -{\mathbf{M}}_{\text{nn}}^{-1}{\mathbf{P}}_{\text{nc}}& {\mathbf{M}}_{\text{nn}}^{-1}\end{array}\right]$$

where $\text{Var}\left({\varvec{\upxi}}\right)={\mathbf{M}}_{\text{nn}}={\mathbf{G}}_{\text{nn}}-{{\mathbf{G}}_{\text{nc}}{\mathbf{G}}_{\text{cc}}^{-1}\mathbf{G}}_{\text{cn}}$. In practice, and hereafter, ${\mathbf{M}}_{\text{nn}}$ is assumed to be a diagonal matrix, that is, ${\mathbf{M}}_{\text{nn}}=\text{diag}\left({\mathbf{G}}_{\text{nn}}-{{\mathbf{G}}_{\text{nc}}{\mathbf{G}}_{\text{cc}}^{-1}\mathbf{G}}_{\text{cn}}\right)$ [9]. This implies that, conditional on core animals, the breeding values of non-core animals are conditionally independent, which is more explicitly shown in [8], and is a variant of the so-called “approximate kernel methods” [18, 19]. It has to be noted that, even when $\mathbf{G}$ is full rank and invertible, ${\mathbf{G}}_{\text{APY}}^{-1}$ is not equal to ${\mathbf{G}}^{-1}$.

### APY-GBLUP

Based on the matrices from Eq. (3), the APY-GBLUP model is defined as:

$$\mathbf{y}=\mathbf{X}\mathbf{b}+\mathbf{W}\mathbf{u}+\mathbf{e},$$

$$\text{E}\left[\mathbf{y}\right]=\mathbf{X}\mathbf{b},$$

4 $$\text{Var}\left[\begin{array}{c}\mathbf{u}\\ \mathbf{e}\end{array}\right]=\left[\begin{array}{cc}{\mathbf{G}}_{\text{APY}}{\upsigma }_{\text{u}}^{2}& \boldsymbol{0}\\ \boldsymbol{0}& \mathbf{I}{\upsigma }_{\text{e}}^{2}\end{array}\right],$$

where $\mathbf{y}$ is the vector of phenotypes, $\mathbf{b}$ is the vector of fixed effects, $\mathbf{e}$ is the vector of error terms, $\mathbf{X}$ and $\mathbf{W}$ are design matrices, and ${\upsigma }_{\text{u}}^{2}$ and ${\upsigma }_{\text{e}}^{2}$ are the genetic and residual variances, respectively. Hereafter, it will be assumed that $\text{E}\left[\mathbf{y}\right]=\mathbf{X}\mathbf{b}$. Assuming multivariate normality for $\mathbf{u}$ and $\mathbf{e}$, the MME for the APY-GBLUP model are:

5 $$\left[\begin{array}{cc}\mathbf{X}\mathbf{^{\prime}}\mathbf{X}& \mathbf{X}\mathbf{^{\prime}}\mathbf{W}\\ \mathbf{W}\mathbf{^{\prime}}\mathbf{X}& \mathbf{W}\mathbf{^{\prime}}\mathbf{W}+{\mathbf{G}}_{\text{APY}}^{-1}\alpha \end{array}\right]\left[\begin{array}{c}\widehat{\mathbf{b}}\\ \widehat{\mathbf{u}}\end{array}\right]=\left[\begin{array}{c}\mathbf{X}\mathbf{^{\prime}}\mathbf{y}\\ \mathbf{W}\mathbf{^{\prime}}\mathbf{y}\end{array}\right],$$

where $$\alpha =\frac{{\upsigma }_{\text{e}}^{2}}{{\upsigma }_{\text{u}}^{2}}$$.

Holding its expectation and covariance structure, the model of Eq. (4) can be partitioned into core and non-core animals as follows:

6 $$\left[\begin{array}{c}{\mathbf{y}}_{\text{c}}\\ {\mathbf{y}}_{\text{n}}\end{array}\right]=\left[\begin{array}{c}{\mathbf{X}}_{\text{c}}\\ {\mathbf{X}}_{\text{n}}\end{array}\right]\mathbf{b}+\left[\begin{array}{cc}{\mathbf{W}}_{\text{c}}& \boldsymbol{0}\\ \boldsymbol{0}& {\mathbf{W}}_{\text{n}}\end{array}\right]\left[\begin{array}{c}{\mathbf{u}}_{\text{c}}\\ {\mathbf{u}}_{\text{n}}\end{array}\right]+\mathbf{e},$$

resulting in the following MME:

7 $$\left[\begin{array}{ccc}{\mathbf{X}}{\mathbf{^{\prime}}}{\mathbf{X}}& {\mathbf{X}}_{\mathrm{c}}^{{^{\prime}}}{\mathbf{W}}_{\mathrm{c}}& {\mathbf{X}}_{\mathrm{n}}^{{^{\prime}}}{\mathbf{W}}_{\mathrm{n}}\\ {\mathbf{W}}_{\mathrm{c}}^{{^{\prime}}}{\mathbf{X}}_{\mathrm{c}}&{\mathbf{W}}_{\mathrm{c}}^{{^{\prime}}}{\mathbf{W}}_{\mathrm{c}}+{\mathbf{G}}_{\mathrm{APY}}^{\mathrm{cc}}\alpha &{\mathbf{G}}_{\mathrm{APY}}^{\mathrm{cn}}\alpha \\ {\mathbf{W}}_{\mathrm{n}}^{{^{\prime}}}{\mathbf{X}}_{\mathrm{n}}& {\mathbf{G}}_{\mathrm{APY}}^{\mathrm{nc}}\alpha & {\mathbf{W}}_{\mathrm{n}}^{{^{\prime}}}{\mathbf{W}}_{\mathrm{n}}+{\mathbf{G}}_{\mathrm{APY}}^{\mathrm{nn}}\alpha \end{array}\right]\left[\begin{array}{c}\widehat{\mathbf{b}}\\ {\widehat{\mathbf{u}}}_{\mathrm{c}}\\ {\widehat{\mathbf{u}}}_{\mathrm{n}}\end{array}\right]=\left[\begin{array}{c}\mathbf{X}\mathbf{^{\prime}}\mathbf{y}\\ {\mathbf{W}}_{\mathrm{c}}^{{^{\prime}}}{\mathbf{y}}_{\mathrm{c}}\\ {\mathbf{W}}_{\mathrm{n}}^{{^{\prime}}}{\mathbf{y}}_{\mathrm{n}}\end{array}\right],$$

where the superscripts in ${\mathbf{G}}_{\text{APY}}$ denote the blocks of ${\mathbf{G}}_{\text{APY}}^{-1}$ corresponding to a specific combination of core and non-core animals. Substituting ${\mathbf{u}}_{\text{n}}={\mathbf{P}}_{\text{nc}}{\mathbf{u}}_{\text{c}}+{\varvec{\upxi}}$ in Eq. (6) leads to the following model:

$$\left[\begin{array}{c}{\mathbf{y}}_{\text{c}}\\ {\mathbf{y}}_{\text{n}}\end{array}\right]=\left[\begin{array}{c}{\mathbf{X}}_{\text{c}}\\ {\mathbf{X}}_{\text{n}}\end{array}\right]\mathbf{b}+\left[\begin{array}{c}{\mathbf{W}}_{\text{c}}\\ {\mathbf{W}}_{\text{n}}{\mathbf{P}}_{\text{nc}}\end{array}\right]{\mathbf{u}}_{\text{c}}+\left[\begin{array}{c}\boldsymbol{0}\\ {\mathbf{W}}_{\text{n}}\end{array}\right]{\varvec{\upxi}}+\mathbf{e},$$

8 $$\text{Var}\left[\begin{array}{c}{\mathbf{u}}_{\text{c}}\\ {\varvec{\upxi}}\\ \mathbf{e}\end{array}\right]=\left[\begin{array}{ccc}{\mathbf{G}}_{\text{cc}}{\upsigma }_{\text{u}}^{2}& \boldsymbol{0}& \boldsymbol{0}\\ \boldsymbol{0}& {\mathbf{M}}_{\text{nn}}{\upsigma }_{\text{u}}^{2}& \boldsymbol{0}\\ \boldsymbol{0}& \boldsymbol{0}& \mathbf{I}{\upsigma }_{\text{e}}^{2}\end{array}\right],$$

and the following MME:

9 $$\left[\begin{array}{ccc}\mathbf{X}^{\prime}\mathbf{X}& {\mathbf{X}}_{\text{c}}^{\prime}{\mathbf{W}}_{\text{c}}& {\mathbf{X}}_{\text{n}}^{\prime}{\mathbf{W}}_{\text{n}}\\ {\mathbf{W}}_{\text{c}}^{\prime}{\mathbf{X}}_{\text{c}}& {\mathbf{W}}_{\text{c}}^{\prime}{\mathbf{W}}_{\text{c}}+{\mathbf{P}}_{\text{cn}}{\mathbf{W}}_{\text{n}}^{\prime}{\mathbf{W}}_{\text{n}}{\mathbf{P}}_{\text{nc}}+{\mathbf{G}}_{\text{cc}}^{-1}\alpha & {\mathbf{P}}_{\text{cn}}{\mathbf{W}}_{\text{n}}^{\prime}{\mathbf{W}}_{\text{n}}\\ {\mathbf{W}}_{\text{n}}^{\prime}{\mathbf{X}}_{\text{n}}& {\mathbf{W}}_{\text{n}}^{\prime}{\mathbf{W}}_{\text{n}}{\mathbf{P}}_{\text{nc}}& {\mathbf{W}}_{\text{n}}^{\prime}{\mathbf{W}}_{\text{n}}+{\mathbf{M}}_{\text{nn}}^{-1}\alpha \end{array}\right]\left[\begin{array}{c}\widehat{\mathbf{b}}\\ {\widehat{\mathbf{u}}}_{\text{c}}\\ \widehat{{\varvec{\upxi}}}\end{array}\right]=\left[\begin{array}{c}\mathbf{X}{^{\prime}}\mathbf{y}\\ {\mathbf{W}}_{\text{c}}^{\prime}{\mathbf{y}}_{\text{c}}\\ {\mathbf{W}}_{\text{n}}^{\prime}{\mathbf{y}}_{\text{n}}\end{array}\right].$$

Then, the BLUP of ${\mathbf{u}}_{\text{n}}$ is obtained as ${\widehat{\mathbf{u}}}_{\text{n}}={\mathbf{P}}_{\text{nc}}{\widehat{\mathbf{u}}}_{\text{c}}+\widehat{{\varvec{\upxi}}}$. These MME are similar to those shown in Eq. (13) in [12] but differ in the error term ${\varvec{\upxi}}$.

### An equivalent model: APY-SNP-BLUP

Assuming that the genetic value of the core animals is fully explained by the markers, then:

$${\mathbf{u}}_{\text{c}}={\mathbf{Z}}_{\text{c}}\mathbf{a}$$ and

10 $${\mathbf{u}}_{\text{n}}={\mathbf{Z}}_{\text{n}}{\mathbf{Z}}_{\text{c}}^{\prime}{\left({\mathbf{Z}}_{\text{c}}{\mathbf{Z}}_{\text{c}}^{\prime}\right)}^{-1}{\mathbf{Z}}_{\text{c}}\mathbf{a}+{\varvec{\upxi}}={\mathbf{Z}}_{\text{n}}\varvec{\mathcal{P}}\mathbf{a}+{\varvec{\upxi}},$$

where $\mathbf{a}$ is the vector of marker effects, and $\varvec{\mathcal{P}}={\mathbf{Z}}_{\text{c}}^{\prime}{\left({\mathbf{Z}}_{\text{c}}{\mathbf{Z}}_{\text{c}}^{\prime}\right)}^{-1}{\mathbf{Z}}_{\text{c}}$, that is, $\varvec{\mathcal{P}}$ is the perpendicular projection operator [11] to $\varvec{\mathcal{C}}\left({\mathbf{Z}}_{\text{c}}^{\prime}\right)$ (i.e., the vector space spanned by the genotypes of the core animals). Assuming that $k{\upsigma }_{\text{u}}^{2}$ is the variance of marker effects, i.e. $\text{Var}\left(\mathbf{a}\right)=\mathbf{I}k{\upsigma }_{\text{u}}^{2}$, $\text{Var}\left({\varvec{\upxi}}\right)={\mathbf{M}}_{\text{nn}}{\upsigma }_{\text{u}}^{2}$, and $\text{cov}\left(\mathbf{a},{\varvec{\upxi}}\right)=\boldsymbol{0}$. Then:

$$\text{Var}\left({\mathbf{u}}_{\text{c}}\right)={\mathbf{Z}}_{\text{c}}\text{Var}\left(\mathbf{a}\right){\mathbf{Z}}_{\text{c}}^{\prime}={\upsigma }_{\text{u}}^{2}k{\mathbf{Z}}_{\text{c}}{\mathbf{Z}}_{\text{c}}^{\prime}$$ and

11 $$\text{Var}\left({\mathbf{u}}_{\text{n}}\right)={\mathbf{Z}}_{\text{n}}\varvec{\mathcal{P}}\text{Var}\left(\mathbf{a}\right)\varvec{\mathcal{P}}{\mathbf{Z}}_{\text{n}}^{\prime}+\text{Var}\left({\varvec{\upxi}}\right)={\upsigma }_{\text{u}}^{2}k{\mathbf{Z}}_{\text{n}}\varvec{\mathcal{P}}{\mathbf{Z}}_{\text{n}}^{\prime}+{\upsigma }_{\text{u}}^{2}{\mathbf{M}}_{\text{nn}}.$$

Letting $${\mathbf{Z}}^{\dagger}=\left[\begin{array}{c}{\mathbf{Z}}_{\text{c}}\\ {\mathbf{Z}}_{\text{n}}\varvec{\mathcal{P}}\end{array}\right]$$ and $$\mathbf{Q}=\left[\begin{array}{c}\boldsymbol{0}\\ {\mathbf{W}}_{\text{n}}\end{array}\right]$$, the following APY-SNP-BLUP model is equivalent to the APY-GBLUP model presented in Eq. (4):

$$\mathbf{y}=\mathbf{X}\mathbf{b}+\mathbf{W}{\mathbf{Z}}^{\dagger}\mathbf{a}+\mathbf{Q}{\varvec{\upxi}}+\mathbf{e}$$, with

12 $$\text{Var}\left[\begin{array}{c}\mathbf{a}\\ {\varvec{\upxi}}\\ \mathbf{e}\end{array}\right]=\left[\begin{array}{ccc}k\mathbf{I}{\upsigma }_{\text{u}}^{2}& \boldsymbol{0}& \boldsymbol{0}\\ \boldsymbol{0}& {\mathbf{M}}_{\text{nn}}{\upsigma }_{\text{u}}^{2}& \boldsymbol{0}\\ \boldsymbol{0}& \boldsymbol{0}& \mathbf{I}{\upsigma }_{\text{e}}^{2}\end{array}\right].$$

Assuming multivariate normality for $\mathbf{a}$, ${\varvec{\upxi}}$, and $\mathbf{e}$, the MME for the APY-SNP-BLUP model are:

13 $$\left[\begin{array}{ccc}\mathbf{X}\mathbf{^{\prime}}\mathbf{X}& \mathbf{X}\mathbf{^{\prime}}\mathbf{W}{\mathbf{Z}}^{\dagger}& {\mathbf{X}}_{\text{n}}^{\prime}{\mathbf{W}}_{\text{n}}\\ {\mathbf{Z}}^{{\dagger}^{\mathbf{^{\prime}}}}\mathbf{W}\mathbf{^{\prime}}\mathbf{X}& {\mathbf{Z}}^{{\dagger}^{\mathbf{^{\prime}}}}\mathbf{W}\mathbf{^{\prime}}\mathbf{W}{\mathbf{Z}}^{\dagger}+\mathbf{I}\gamma & \varvec{\mathcal{P}}{\mathbf{Z}}_{\text{n}}^{\prime}{\mathbf{W}}_{\text{n}}^{\prime}{\mathbf{W}}_{\text{n}}\\ {\mathbf{W}}_{\text{n}}^{\prime}{\mathbf{X}}_{\text{n}}& {\mathbf{W}}_{\text{n}}^{\prime}{\mathbf{W}}_{\text{n}}{\mathbf{Z}}_{\text{n}}\varvec{\mathcal{P}}& {\mathbf{W}}_{\text{n}}^{\prime}{\mathbf{W}}_{\text{n}}+{\mathbf{M}}_{\text{nn}}^{-1}\alpha \end{array}\right]\left[\begin{array}{c}\widehat{\mathbf{b}}\\ \widehat{\mathbf{a}}\\ \widehat{{\varvec{\upxi}}}\end{array}\right]=\left[\begin{array}{c}\mathbf{X}\mathbf{^{\prime}}\mathbf{y}\\ {\mathbf{Z}}^{{\dagger}^{\mathbf{^{\prime}}}}\mathbf{W}\mathbf{^{\prime}}\mathbf{y}\\ {\mathbf{W}}_{\text{n}}^{\prime}{\mathbf{y}}_{\text{n}}\end{array}\right],$$

where $$\gamma =\frac{{\upsigma }_{\text{e}}^{2}}{k{\upsigma }_{\text{u}}^{2}}$$.

If $rank\left({\mathbf{Z}}_{\text{c}}\right)=rank\left(\mathbf{Z}\right)$, which is true when the number of core animals is equal to the number of markers and given a non-singular ${\mathbf{G}}_{\text{cc}}$, $\varvec{\mathcal{P}}=\mathbf{I}$. Further, ${\mathbf{M}}_{\text{nn}}=\boldsymbol{0}$, and since ${\varvec{\upxi}}$ has null expectation, ${\varvec{\upxi}}=\boldsymbol{0}$. Consequently, for a sufficiently large number of core animals:

$${\mathbf{u}}_{\text{c}}={\mathbf{Z}}_{\text{c}}\mathbf{a}$$ and

14 $${\mathbf{u}}_{\text{n}}={\mathbf{Z}}_{\text{n}}\varvec{\mathcal{P}}\mathbf{a}+{\varvec{\upxi}}={\mathbf{Z}}_{\text{n}}\mathbf{a}+{\varvec{\upxi}}={\mathbf{Z}}_{\text{n}}\mathbf{a}.$$

Then, the model in Eq. (12) reduces to:

$$\mathbf{y}=\mathbf{X}\mathbf{b}+\mathbf{W}\mathbf{Z}\mathbf{a}+\mathbf{e},$$

15 $$\text{with \,Var}\left[\begin{array}{c}\mathbf{a}\\ \mathbf{e}\end{array}\right]=\left[\begin{array}{cc}k\mathbf{I}{\upsigma }_{\text{u}}^{2}& \boldsymbol{0}\\ \boldsymbol{0}& \mathbf{I}{\upsigma }_{\text{e}}^{2}\end{array}\right],$$

and the MME are:

16 $$\left[\begin{array}{cc}\mathbf{X}\mathbf{^{\prime}}\mathbf{X}& \mathbf{X}\mathbf{^{\prime}}\mathbf{W}\\ \mathbf{W}\mathbf{^{\prime}}\mathbf{X}& \mathbf{Z}\mathbf{^{\prime}}\mathbf{W}\mathbf{^{\prime}}\mathbf{W}\mathbf{Z}+\mathbf{I}\gamma \end{array}\right]\left[\begin{array}{c}\widehat{\mathbf{b}}\\ \widehat{\mathbf{a}}\end{array}\right]=\left[\begin{array}{c}\mathbf{X}\mathbf{^{\prime}}\mathbf{y}\\ \mathbf{Z}\mathbf{^{\prime}}\mathbf{W}\mathbf{^{\prime}}\mathbf{y}\end{array}\right],$$

which are the MME for a typical SNP-BLUP model [1]. Thus, APY-SNP-BLUP converges to the regular SNP-BLUP when the number of core animals increases.

### Distribution of breeding values and marker effects with APY

By defining the breeding values as in Eq. (10) and keeping the distributional assumptions of $\mathbf{a}$ and ${\varvec{\upxi}}$ from Eq. (12), the covariance matrix of the joint distribution of $\mathbf{u}$ and $\mathbf{a}$ is:

17 $$\text{Var}\left[\begin{array}{c}\mathbf{u}\\ \mathbf{a}\end{array}\right]=\left[\begin{array}{cc}k{\mathbf{Z}}^{\dagger}{\mathbf{Z}}^{{\dagger}^{\boldsymbol{^{\prime}}}}+\mathbf{V}& k{\mathbf{Z}}^{\dagger}\\ k{\mathbf{Z}}^{{\dagger}^{\boldsymbol{^{\prime}}}}& k\mathbf{I}\end{array}\right]{\upsigma }_{\text{u}}^{2},$$

where $$\mathbf{V}=\left[\begin{array}{cc}\boldsymbol{0}& \boldsymbol{0}\\ \boldsymbol{0}& {\mathbf{M}}_{\text{nn}}\end{array}\right]$$. Then, assuming normality for $\mathbf{a}$ and ${\varvec{\upxi}}$, the conditional distribution of $\mathbf{u}$ on $\mathbf{a}$ is:

18 $$p\left(\mathbf{u}|\mathbf{a}\right)=N\left({\mathbf{Z}}^{\dagger}\mathbf{a},\mathbf{V}{\upsigma }_{\text{u}}^{2}\right).$$

Note that this is a degenerate normal distribution because its covariance matrix is non-positive definite. In the classical GBLUP and SNP-BLUP models [1], the variance of $\mathbf{u}$ conditional on $\mathbf{a}$ is zero (i.e., if marker effects are known, the breeding value is fully explained), whereas Eq. (18) shows that in APY, the variance of $\mathbf{u}$ conditional on $\mathbf{a}$ is nonzero for the non-core animals.

Conversely, the conditional distribution of $\mathbf{a}$ on $\mathbf{u}$ is:

19 $$p\left(\mathbf{a}|\mathbf{u}\right)=N\left(k{\mathbf{Z}}^{{\dagger}^{\boldsymbol{^{\prime}}}}{\left(k{\mathbf{Z}}^{\dagger}{\mathbf{Z}}^{{\dagger}^{\boldsymbol{^{\prime}}}}+\mathbf{V}\right)}^{-1}\mathbf{u},\left(k\mathbf{I}-k{\mathbf{Z}}^{{\dagger}^{\boldsymbol{^{\prime}}}}{\left(k{\mathbf{Z}}^{\dagger}{\mathbf{Z}}^{{\dagger}^{\boldsymbol{^{\prime}}}}+\mathbf{V}\right)}^{-1}{\mathbf{Z}}^{\dagger}k\right){\upsigma }_{\text{u}}^{2}\right).$$

The BLUP of $\mathbf{a}$ given $\mathbf{u}=\widehat{\mathbf{u}}$ can be obtained from Eq. (19) as [1]:

20 $$\widehat{\mathbf{a}}|\widehat{\mathbf{u}}=\text{E}\left[\mathbf{a}|\mathbf{u}=\widehat{\mathbf{u}}\right]=k{\mathbf{Z}}^{{\dagger}^{\boldsymbol{^{\prime}}}}{\left(k{\mathbf{Z}}^{\dagger}{\mathbf{Z}}^{{\dagger}^{\boldsymbol{^{\prime}}}}+\mathbf{V}\right)}^{-1}\widehat{\mathbf{u}}=k{\mathbf{Z}}^{{\dagger}^{\boldsymbol{^{\prime}}}}{\mathbf{G}}_{\text{APY}}^{-1}\widehat{\mathbf{u},}$$

which after algebra reduces to [20]:

21 $$\widehat{\mathbf{a}}|\widehat{\mathbf{u}}=k{\mathbf{Z}}^{{\dagger}^{\boldsymbol{^{\prime}}}}{\mathbf{G}}_{\text{APY}}^{-1}\widehat{\mathbf{u}}=k{\mathbf{Z}}_{\text{c}}^{\prime}{\mathbf{G}}_{\text{cc}}^{-1}{\widehat{\mathbf{u}}}_{\text{c}},$$

with variance equal to:

22 $$\text{Var}\left(\widehat{\mathbf{a}}|\widehat{\mathbf{u}}\right)={k}^{2}{\mathbf{Z}}_{\text{c}}^{\prime}{\mathbf{G}}_{\text{cc}}^{-1}\left({\mathbf{G}}_{\text{cc}}-{\text{PEV}}_{\text{core}}\right){\mathbf{G}}_{\text{cc}}^{-1}{\mathbf{Z}}_{\text{c}}.$$

Thus, in order to obtain predictions of marker effects from the APY-GBLUP model, only the estimated breeding values of the core animals and the corresponding blocks of matrices are needed.

### Indirect predictions with APY

Indirect predictions $\left({\widehat{\mathbf{u}}}_{\text{ip}}\right)$ are estimated breeding values for animals without own records or progeny with records, based on estimated marker effects from the genetic evaluation. Therefore, an indirect-predicted animal is by definition a non-core animal. Using Eq. (18), indirect predictions with APY are calculated as:

23 $${\widehat{\mathbf{u}}}_{\text{ip}}|\widehat{\mathbf{a}}={\mathbf{Z}}_{\text{ip}}\varvec{\mathcal{P}}\widehat{\mathbf{a}}={\mathbf{G}}_{\text{ip},\text{c}}{\mathbf{G}}_{\text{cc}}^{-1}{\widehat{\mathbf{u}}}_{\text{c}},$$

where ${\mathbf{Z}}_{\text{ip}}$ is the centered and scaled genotype matrix for the indirect-predicted animals, and ${\mathbf{G}}_{\text{ip},\text{c}}$ is the genomic relationship matrix between indirect-predicted and core animals. Note that the genotype matrix ${\mathbf{Z}}_{\text{ip}}$ has to be centered and scaled in exactly the same manner as the matrices $$\mathbf{Z}=\left[\begin{array}{c}{\mathbf{Z}}_{\text{c}}\\ {\mathbf{Z}}_{\text{n}}\end{array}\right]$$ that are used for genomic evaluation by any method; otherwise, the algebra (i.e. Eq. (17)) used to derive the joint distribution of breeding values and markers is an approximation. Changing the centering of ${\mathbf{Z}}_{\text{ip}}$ (i.e., using a different set of allele frequencies) results in a shift of the mean and, in addition, a different implicit ${\varvec{\upxi}}$ in the APY approximation. Changing the scale of ${\mathbf{Z}}_{\text{ip}}$ (i.e., multiplying by a different $k$) changes the scale of marker effects and of the indirect predictions.

Note that the leftmost expression in Eq. (23) is, as expected, the selection index formulation for estimation of breeding values. Two measures of uncertainty are associated with estimation of ${\widehat{\mathbf{u}}}_{\text{ip}}$. First, the uncertainty associated with the variance in Eq. (18) that arises from the error term in Eq. (10), i.e. due to the approximation in APY. This uncertainty is calculated similarly to a reliability:

24 $${\uprho }_{\text{i}}=1-\frac{\mathbf{m}_{\text{ii}}}{\mathbf{g}_{\text{ii}}},$$

where ${\mathbf{g}}_{\text{ii}}$ and ${\mathbf{m}}_{\text{ii}}$ are the diagonal element of $\mathbf{G}$ and the element of ${\mathbf{M}}_{\text{nn}}$, respectively, corresponding to the $i$^*th*^ animal. Equation (24) converges to one when ${\varvec{\upxi}}\to 0$, which occurs when the size of the core increases. The second measure of uncertainty is the usual reliability associated with the prediction error variance of ${\widehat{\mathbf{u}}}_{\text{ip}}|\widehat{\mathbf{a}}$, which is:

25 $$\text{Var}{\left({\widehat{\mathbf{u}}}_{\text{ip}}|\widehat{\mathbf{a}}-{\mathbf{u}}_{\text{ip}}\right)}_{\text{i}}={\mathbf{m}}_{\text{ii}}+{\mathbf{g}}_{\text{i},\text{c}}{\mathbf{G}}_{\text{cc}}^{-1}{\text{PEV}}_{\text{core}}{\mathbf{G}}_{\text{cc}}^{-1}{\mathbf{g}}_{\text{c},\text{i}},$$

where ${\mathbf{g}}_{\text{i},\text{c}}$ is the block of $\mathbf{G}$ that relates the $i$ ^*th*^ individual with the core animals, and ${\mathbf{g}}_{\text{c},\text{i}}={\mathbf{g}}_{\text{i},\text{c}}\mathbf{^{\prime}}$. The reliability associated with Eq. (25) is:

26 $${\text{rel}}_{\text{i}}=1-\frac{{\mathbf{m}}_{\text{ii}}+{\mathbf{g}}_{\text{i},\text{c}}{\mathbf{G}}_{\text{cc}}^{-1}{\text{PEV}}_{\text{core}}{\mathbf{G}}_{\text{cc}}^{-1}{\mathbf{g}}_{\text{c},\text{i}}}{{\mathbf{g}}_{\text{ii}}}={\uprho }_{\text{i}}-\frac{{\mathbf{g}}_{\text{i},\text{c}}{\mathbf{G}}_{\text{cc}}^{-1}{\text{PEV}}_{\text{core}}{\mathbf{G}}_{\text{cc}}^{-1}{\mathbf{g}}_{\text{c},\text{i}}}{{\mathbf{g}}_{\text{ii}}}.$$

### APY-GBLUP and APY-SNP-BLUP models with a residual polygenic effect

To consider an extra polygenic effect based on pedigree, ${\mathbf{G}}_{\text{APY}}^{-1}$ in Eq. (5) can be calculated based on the following ${\mathbf{G}}^{\mathbf{*}}$:

27 $${\mathbf{G}}^{\mathbf{*}}={\mathbf{Z}}^{\mathbf{*}}{\mathbf{Z}}^{{\mathbf{*}}^{\mathbf{^{\prime}}}}=\left[\begin{array}{cc}\sqrt{\left(1-\beta \right)k}\mathbf{Z}& \sqrt{\beta }\mathbf{L}\end{array}\right]\left[\begin{array}{c}\sqrt{\left(1-\beta \right)k}\mathbf{Z}\mathbf{^{\prime}}\\ \sqrt{\beta }\mathbf{L}\mathbf{^{\prime}}\end{array}\right]=\left(1-\beta \right)\left(k\mathbf{Z}\mathbf{Z}\mathbf{^{\prime}}\right)+\beta {\mathbf{A}}_{22},$$

where ${\mathbf{A}}_{22}$ is the block of the numerator relationship matrix corresponding to the genotyped animals, $\mathbf{L}$ is the Cholesky factor or its approximation of ${\mathbf{A}}_{22}$ [21], and $\beta$ is the proportion of the residual polygenic effect. When the matrices from model Eq. (4) are constructed based on Eq. (27), the resulting model is designated as APY-GBLUP with a residual polygenic effect. In this case, the breeding values are not fully explained by the markers. Therefore:

$${\mathbf{u}}_{\text{c}}={\mathbf{Z}}_{\text{c}}\mathbf{a}+{\varvec{\upvarepsilon}},$$

28 $${\mathbf{u}}_{\text{n}}={\mathbf{P}}_{\text{nc}}^{*}\left({\mathbf{Z}}_{\text{c}}\mathbf{a}+{\varvec{\upvarepsilon}}\right)+{\varvec{\upxi}}={\mathbf{Z}}_{\text{n}}^{*}{\varvec{\mathcal{P}}}^{*}\mathbf{S}\mathbf{a}+{\mathbf{Z}}_{\text{n}}^{*}{\mathbf{Z}}_{\text{c}}^{{*}^{\prime}}{\left({\mathbf{Z}}_{\text{c}}^{*}{\mathbf{Z}}_{\text{c}}^{{*}^{\prime}}\right)}^{-1}{\varvec{\upvarepsilon}}+{\varvec{\upxi}},$$

where ${\varvec{\upvarepsilon}}\sim \text{MVN}\left(\boldsymbol{0},\beta {\mathbf{A}}_{\text{cc}}{\upsigma }_{\text{u}}^{2}\right)$ is the vector of residual polygenic effects [22], ${\mathbf{A}}_{\text{cc}}$ is the block of the numerator relationship matrix corresponding to the core animals, ${\mathbf{P}}_{\text{nc}}^{*}={\mathbf{G}}_{\text{nc}}^{\mathbf{*}}{\mathbf{G}}_{\text{cc}}^{{\mathbf{*}}^{-1}}$, ${\varvec{\mathcal{P}}}^{*}={\mathbf{Z}}_{\text{c}}^{{*}^{\prime}}{\left({\mathbf{Z}}_{\text{c}}^{*}{\mathbf{Z}}_{\text{c}}^{{*}^{\prime}}\right)}^{-1}{\mathbf{Z}}_{\text{c}}^{*}$, and $$\mathbf{S}=\left[\begin{array}{c}\mathbf{I}\frac{1}{\sqrt{\left(1-\beta \right)k}}\\ \boldsymbol{0}\end{array}\right]$$. Then, a marker effects model equivalent to APY-GBLUP with a residual polygenic effect is:

$$\mathbf{y}=\mathbf{X}\mathbf{b}+\mathbf{W}{\mathbf{Z}}^{\dagger\mathbf{*}}\mathbf{a}+\mathbf{W}\mathbf{R}{\varvec{\upvarepsilon}}+\mathbf{Q}{\varvec{\upxi}}+\mathbf{e},$$

29 $$\text{Var}\left[\begin{array}{c}\mathbf{a}\\ {\varvec{\upvarepsilon}}\\ {\varvec{\upxi}}\\ \mathbf{e}\end{array}\right]=\left[\begin{array}{cccc}\left(1-\beta \right)k\mathbf{I}{\upsigma }_{\text{u}}^{2}& \boldsymbol{0}& \boldsymbol{0}& \boldsymbol{0}\\ \boldsymbol{0}& \beta {\mathbf{A}}_{\text{cc}}{\upsigma }_{\text{u}}^{2}& \boldsymbol{0}& \boldsymbol{0}\\ \boldsymbol{0}& \boldsymbol{0}& {\mathbf{M}}_{\text{nn}}{\upsigma }_{\text{u}}^{2}& \boldsymbol{0}\\ \boldsymbol{0}& \boldsymbol{0}& \boldsymbol{0}& \mathbf{I}{\upsigma }_{\text{e}}^{2}\end{array}\right],$$

where $${\mathbf{Z}}^{\dagger\mathbf{*}}=\left[\begin{array}{c}{\mathbf{Z}}_{\text{c}}\\ {\mathbf{Z}}_{\text{n}}^{*}{\varvec{\mathcal{P}}}^{*}\mathbf{S}\end{array}\right]$$ and $$\mathbf{R}=\left[\begin{array}{c}\mathbf{I}\\ {\mathbf{Z}}_{\text{n}}^{*}{\mathbf{Z}}_{\text{c}}^{{*}^{\prime}}{\left({\mathbf{Z}}_{\text{c}}^{*}{\mathbf{Z}}_{\text{c}}^{{*}^{\prime}}\right)}^{-1}\end{array}\right]$$. The MME for the model in Eq. (29) are:

30 $$\left[\begin{array}{cccc}\mathbf{X}\mathbf{^{\prime}}\mathbf{X}& \mathbf{X}\mathbf{^{\prime}}\mathbf{W}{\mathbf{Z}}^{\dagger\mathbf{*}}& \mathbf{X}\mathbf{^{\prime}}\mathbf{W}\mathbf{T}& {\mathbf{X}}_{\text{n}}^{\prime}{\mathbf{W}}_{\text{n}}\\ {\mathbf{Z}}^{{\dagger\mathbf{*}}^{\mathbf{^{\prime}}}}\mathbf{W}\mathbf{^{\prime}}\mathbf{X}& {\mathbf{Z}}^{{\dagger\mathbf{*}}^{\mathbf{^{\prime}}}}\mathbf{W}\mathbf{^{\prime}}\mathbf{W}{\mathbf{Z}}^{\dagger\mathbf{*}}+\mathbf{I}\delta & {\mathbf{Z}}^{{\dagger\mathbf{*}}^{\mathbf{^{\prime}}}}\mathbf{W}\mathbf{^{\prime}}\mathbf{W}\mathbf{T}& \mathbf{S}\mathbf{^{\prime}}{\varvec{\mathcal{P}}}^{*}{\mathbf{Z}}_{\text{n}}^{{*}^{\prime}}{\mathbf{W}}_{\text{n}}^{\prime}{\mathbf{W}}_{\text{n}}\\ \mathbf{T}\mathbf{^{\prime}}\mathbf{W}\mathbf{^{\prime}}\mathbf{X}& \mathbf{T}\mathbf{^{\prime}}\mathbf{W}\mathbf{^{\prime}}\mathbf{W}{\mathbf{Z}}^{\dagger\mathbf{*}}& \mathbf{T}\mathbf{^{\prime}}\mathbf{W}\mathbf{^{\prime}}\mathbf{W}\mathbf{T}+{\mathbf{A}}_{\text{cc}}^{-1}\zeta & {\left({\mathbf{Z}}_{\text{c}}^{*}{\mathbf{Z}}_{\text{c}}^{{*}^{\prime}}\right)}^{-1}{\mathbf{Z}}_{\text{c}}^{*}{\mathbf{Z}}_{\text{n}}^{{*}^{\prime}}{\mathbf{W}}_{\text{n}}^{\prime}{\mathbf{W}}_{\text{n}}\\ {\mathbf{W}}_{\text{n}}^{\prime}{\mathbf{X}}_{\text{n}}& {\mathbf{W}}_{\text{n}}^{\prime}{\mathbf{W}}_{\text{n}}{\mathbf{Z}}_{\text{n}}^{*}{\varvec{\mathcal{P}}}^{*}\mathbf{S}& {\mathbf{W}}_{\text{n}}^{\prime}{\mathbf{W}}_{\text{n}}{\mathbf{Z}}_{\text{n}}^{*}{\mathbf{Z}}_{\text{c}}^{{*}^{\prime}}{\left({\mathbf{Z}}_{\text{c}}^{*}{\mathbf{Z}}_{\text{c}}^{{*}^{\prime}}\right)}^{-1}& {\mathbf{W}}_{\text{n}}^{\prime}{\mathbf{W}}_{\text{n}}+{\mathbf{M}}_{\text{nn}}^{-1}\alpha \end{array}\right]\left[\begin{array}{c}\widehat{\mathbf{b}}\\ \widehat{\mathbf{a}}\\ \widehat{{\varvec{\upvarepsilon}}}\\ \widehat{{\varvec{\upxi}}}\end{array}\right]=\left[\begin{array}{c}\mathbf{X}\mathbf{^{\prime}}\mathbf{y}\\ {\mathbf{Z}}^{{\dagger\mathbf{*}}^{\mathbf{^{\prime}}}}\mathbf{W}\mathbf{^{\prime}}\mathbf{y}\\ \mathbf{R}\mathbf{^{\prime}}\mathbf{W}\mathbf{^{\prime}}\mathbf{y}\\ {\mathbf{W}}_{\text{n}}^{\prime}{\mathbf{y}}_{\text{n}}\end{array}\right].$$

where $$\delta =\frac{{\upsigma }_{\text{e}}^{2}}{\left(1-\beta \right)k{\upsigma }_{\text{u}}^{2}}$$ and $$\zeta =\frac{{\upsigma }_{\text{e}}^{2}}{\beta {\upsigma }_{\text{u}}^{2}}$$.

### APY single-step GBLUP (APY-ssGBLUP) and APY single-step SNP-BLUP (APY-ssSNP-BLUP)

For a general ssGBLUP model [23] with APY [14], assuming a genetic variance equal to 1, the covariance matrix of the breeding values is equal to:

31 $$\text{Var}\left[\begin{array}{c}{\mathbf{u}}_{1}\\ {\mathbf{u}}_{2}\end{array}\right]={\mathbf{H}}_{\text{APY}}=\left[\begin{array}{cc}{\mathbf{A}}_{11}+{\mathbf{A}}_{12}{\mathbf{A}}_{22}^{-1}\left({\mathbf{G}}_{\text{APY}}-{\mathbf{A}}_{22}\right){\mathbf{A}}_{22}^{-1}{\mathbf{A}}_{21}& {\mathbf{A}}_{12}{\mathbf{A}}_{22}^{-1}{\mathbf{G}}_{\text{APY}}\\ {\mathbf{G}}_{\text{APY}}{\mathbf{A}}_{22}^{-1}{\mathbf{A}}_{21}& {\mathbf{G}}_{\text{APY}}\end{array}\right],$$

where the subscripts 1 and 2 denote the non-genotyped and genotyped animals, respectively. Letting $${\mathbf{A}}_{22}^{-1}=\left[\begin{array}{cc}{\mathbf{A}}_{22}^{\text{cc}}& {\mathbf{A}}_{22}^{\text{cn}}\\ {\mathbf{A}}_{22}^{\text{nc}}& {\mathbf{A}}_{22}^{\text{nn}}\end{array}\right]$$, the inverse of ${\mathbf{H}}_{\text{APY}}$ is equal to [24]:

32 $$\begin{aligned}{\mathbf{H}}_{\text{APY}}^{-1}&={\mathbf{A}}^{-1}+\left[\begin{array}{cc}\boldsymbol{0}&\boldsymbol{0}\\ \boldsymbol{0}&{\mathbf{G}}_{\text{APY}}^{-1}-{\mathbf{A}}_{22}^{-1}\end{array}\right]=\left[\begin{array}{ccc}{\mathbf{A}}^{11}&{\mathbf{A}}^{1\text{c}}& {\mathbf{A}}^{1\text{n}}\\ {\mathbf{A}}^{\text{c}1}& {\mathbf{A}}^{\text{cc}}&{\mathbf{A}}^{\text{cn}}\\ {\mathbf{A}}^{\text{n}1}& {\mathbf{A}}^{\text{nc}}&{\mathbf{A}}^{\text{nn}}\end{array}\right]+\left[\begin{array}{ccc}\boldsymbol{0}&\boldsymbol{0}& \boldsymbol{0}\\ \boldsymbol{0}&{\mathbf{G}}_{\text{cc}}^{-1}+{\mathbf{P}}_{\text{cn}}{\mathbf{M}}_{\text{nn}}^{-1}{\mathbf{P}}_{\text{nc}}-{\mathbf{A}}_{22}^{\text{cc}}& -{\mathbf{P}}_{\text{cn}}{\mathbf{M}}_{\text{nn}}^{-1}-{\mathbf{A}}_{22}^{\text{cn}}\\ \boldsymbol{0}&-{\mathbf{M}}_{\text{nn}}^{-1}{\mathbf{P}}_{\text{nc}}-{\mathbf{A}}_{22}^{\text{nc}}&{\mathbf{M}}_{\text{nn}}^{-1}-{\mathbf{A}}_{22}^{\text{nn}}\end{array}\right]\end{aligned}.$$

Then, the APY-ssGBLUP model is defined as:

$$\mathbf{y}=\mathbf{X}\mathbf{b}+\mathbf{W}\mathbf{u}+\mathbf{e},$$

33 $$\text{Var}\left[\begin{array}{c}\mathbf{u}\\ \mathbf{e}\end{array}\right]=\left[\begin{array}{cc}{\mathbf{H}}_{\text{APY}}{\upsigma }_{\text{u}}^{2}& \boldsymbol{0}\\ \boldsymbol{0}& \mathbf{I}{\upsigma }_{\text{e}}^{2}\end{array}\right].$$

The MME for the model in Eq. (33) are:

34 $$\left[\begin{array}{ccc}\mathbf{X}\mathbf{^{\prime}}\mathbf{X}& {\mathbf{X}}_{1}^{\prime}{\mathbf{W}}_{1}& {\mathbf{X}}_{2}^{\prime}{\mathbf{W}}_{2}\\ {\mathbf{W}}_{1}^{\prime}{\mathbf{X}}_{1}& {\mathbf{W}}_{1}^{\prime}{\mathbf{W}}_{1}+{\mathbf{A}}^{11}\alpha & {\mathbf{A}}^{12}\alpha \\ {\mathbf{W}}_{\text{c}}^{\prime}{\mathbf{X}}_{\text{c}}& {\mathbf{A}}^{21}\alpha & {\mathbf{W}}_{2}^{\prime}{\mathbf{W}}_{2}+\left({\mathbf{A}}^{22}+{\mathbf{G}}_{\text{APY}}^{-1}-{\mathbf{A}}_{22}^{-1}\right)\alpha \end{array}\right]\left[\begin{array}{c}\widehat{\mathbf{b}}\\ {\widehat{\mathbf{u}}}_{1}\\ {\widehat{\mathbf{u}}}_{2}\end{array}\right]=\left[\begin{array}{c}\mathbf{X}\mathbf{^{\prime}}\mathbf{y}\\ {\mathbf{W}}_{1}^{\prime}{\mathbf{y}}_{1}\\ {\mathbf{W}}_{2}^{\prime}{\mathbf{y}}_{2}\end{array}\right].$$

Following [25], the breeding values of non-genotyped animals can be written as: ${\mathbf{u}}_{1}={\mathbf{A}}_{12}{\mathbf{A}}_{22}^{-1}{\mathbf{u}}_{2}+{\varvec{\upeta}}$, where ${\varvec{\upeta}}\sim \text{MVN}\left(0,{\left({\mathbf{A}}^{11}\right)}^{-1}\right)$ and represents the imputation error [23, 25]. Then, the breeding values of all animals can be expressed as:

35 $$\left[\begin{array}{c}{\mathbf{u}}_{1}\\ {\mathbf{u}}_{\text{c}}\\ {\mathbf{u}}_{\text{n}}\end{array}\right]=\left[\begin{array}{c}{\mathbf{A}}_{12}{\mathbf{A}}_{22}^{-1}\left[\begin{array}{c}{\mathbf{Z}}_{\text{c}}\mathbf{a}\\ {\mathbf{Z}}_{\text{n}}\varvec{\mathcal{P}}\mathbf{a}+{\varvec{\upxi}}\end{array}\right]+{\varvec{\upeta}}\\ {\mathbf{Z}}_{\text{c}}\mathbf{a}\\ {\mathbf{Z}}_{\text{n}}\varvec{\mathcal{P}}\mathbf{a}+{\varvec{\upxi}}\end{array}\right].$$

Letting $${\mathbf{W}}^{\dagger}=\left[\begin{array}{c}{\mathbf{W}}_{1}{\mathbf{A}}_{12}{\mathbf{A}}_{22}^{-1}{\mathbf{Z}}^{\dagger}\\ {\mathbf{W}}_{2}{\mathbf{Z}}^{\dagger}\end{array}\right]$$, $$\mathbf{T}=\left[\begin{array}{c}{\mathbf{W}}_{1}\\ \boldsymbol{0}\end{array}\right]$$, and $${\mathbf{Q}}^{\dagger}=\left[\begin{array}{c}{\mathbf{W}}_{1}{\mathbf{A}}_{12}{\mathbf{A}}_{22}^{-1}\mathbf{Q}\\ \mathbf{Q}\end{array}\right]$$, an equivalent model to that presented in Eq. (33) is:

$$\mathbf{y}=\mathbf{X}\mathbf{b}+{\mathbf{W}}^{\dagger}\mathbf{a}+\mathbf{T}{\varvec{\upeta}}+{\mathbf{Q}}^{\dagger}{\varvec{\upxi}}+\mathbf{e},$$

36 $$\text{Var}\left[\begin{array}{c}\mathbf{a}\\ {\varvec{\upeta}}\\ {\varvec{\upxi}}\\ \mathbf{e}\end{array}\right]=\left[\begin{array}{cccc}k\mathbf{I}{\upsigma }_{\text{u}}^{2}& \boldsymbol{0}& \boldsymbol{0}& \boldsymbol{0}\\ \boldsymbol{0}& {\left({\mathbf{A}}^{11}\right)}^{-1}{\upsigma }_{\text{u}}^{2}& \boldsymbol{0}& \boldsymbol{0}\\ \boldsymbol{0}& \boldsymbol{0}& {\mathbf{M}}_{\text{nn}}{\upsigma }_{\text{u}}^{2}& \boldsymbol{0}\\ \boldsymbol{0}& \boldsymbol{0}& \boldsymbol{0}& \mathbf{I}{\upsigma }_{\text{e}}^{2}\end{array}\right]$$

The MME corresponding to this model are:

37 $$\left[\begin{array}{cccc}\mathbf{X}\mathbf{^{\prime}}\mathbf{X}& \mathbf{X}\mathbf{^{\prime}}{\mathbf{W}}^{\dagger}& {\mathbf{X}}_{1}^{\prime}{\mathbf{W}}_{1}& \mathbf{X}\mathbf{^{\prime}}{\mathbf{Q}}^{\dagger}\\ {\mathbf{W}}^{{\dagger}^{\mathbf{^{\prime}}}}\mathbf{X}& {\mathbf{W}}^{{\dagger}^{\mathbf{^{\prime}}}}{\mathbf{W}}^{\dagger}+\mathbf{I}\gamma & {\mathbf{W}}^{{\dagger}^{\mathbf{^{\prime}}}}\mathbf{T}& {\mathbf{W}}^{{\dagger}^{\mathbf{^{\prime}}}}{\mathbf{Q}}^{\dagger}\\ {\mathbf{W}}_{1}^{\prime}{\mathbf{X}}_{1}& {\mathbf{T}}^{\mathbf{^{\prime}}}{\mathbf{W}}^{\dagger}& {\mathbf{W}}_{1}^{\prime}{\mathbf{W}}_{1}+{\mathbf{A}}^{11}\alpha & {\mathbf{T}}^{\mathbf{^{\prime}}}{\mathbf{Q}}^{\dagger}\\ {\mathbf{Q}}^{{\dagger}^{\mathbf{^{\prime}}}}\mathbf{X}& {\mathbf{Q}}^{{\dagger}^{\mathbf{^{\prime}}}}{\mathbf{W}}^{\dagger}& {\mathbf{Q}}^{{\dagger}^{\mathbf{^{\prime}}}}\mathbf{T}& {\mathbf{Q}}^{{\dagger}^{\mathbf{^{\prime}}}}{\mathbf{Q}}^{\dagger}+{\mathbf{M}}_{\text{nn}}^{-1}\alpha \end{array}\right]\left[\begin{array}{c}\widehat{\mathbf{b}}\\ \widehat{\mathbf{a}}\\ \widehat{{\varvec{\upeta}}}\\ \widehat{{\varvec{\upxi}}}\end{array}\right]=\left[\begin{array}{c}\mathbf{X}\mathbf{^{\prime}}\mathbf{y}\\ {\mathbf{W}}^{{\dagger}^{\mathbf{^{\prime}}}}\mathbf{y}\\ {\mathbf{T}}^{\mathbf{^{\prime}}}\mathbf{y}\\ {\mathbf{Q}}^{{\dagger}^{\mathbf{^{\prime}}}}\mathbf{y}\end{array}\right]$$

### APY-ssGBLUP and APY-ssSNP-BLUP with a residual polygenic effect

If ${\mathbf{G}}_{\text{APY}}^{-1}$ in ${\mathbf{H}}_{\text{APY}}^{-1}$ is built using ${\mathbf{G}}^{\mathbf{*}}$ from Eq. (27), the resulting model is called APY-ssGBLUP with a residual polygenic effect. In such a case, Eq. (35) is modified to:

38 $$\left[\begin{array}{c}{\mathbf{u}}_{1}\\ {\mathbf{u}}_{\text{c}}\\ {\mathbf{u}}_{\text{n}}\end{array}\right]=\left[\begin{array}{c}{\mathbf{A}}_{12}{\mathbf{A}}_{22}^{-1}\left[\begin{array}{c}{\mathbf{Z}}_{\text{c}}\mathbf{a}+{\varvec{\upvarepsilon}}\\ {\mathbf{P}}_{\text{nc}}^{*}\left({\mathbf{Z}}_{\text{c}}\mathbf{a}+{\varvec{\upvarepsilon}}\right)+{\varvec{\upxi}}\end{array}\right]+{\varvec{\upeta}}\\ {\mathbf{Z}}_{\text{c}}\mathbf{a}+{\varvec{\upvarepsilon}}\\ {\mathbf{P}}_{\text{nc}}^{*}\left({\mathbf{Z}}_{\text{c}}\mathbf{a}+{\varvec{\upvarepsilon}}\right)+{\varvec{\upxi}}\end{array}\right]=\left[\begin{array}{c}{\mathbf{A}}_{12}{\mathbf{A}}_{22}^{-1}\left({\mathbf{Z}}^{\dagger\mathbf{*}}\mathbf{a}+\mathbf{R}{\varvec{\upvarepsilon}}+\mathbf{Q}{\varvec{\upxi}}\right)+{\varvec{\upeta}}\\ {\mathbf{Z}}_{\text{c}}\mathbf{a}+{\varvec{\upvarepsilon}}\\ {\mathbf{Z}}_{\text{n}}^{*}{\varvec{\mathcal{P}}}^{*}\mathbf{S}\mathbf{a}+{\mathbf{Z}}_{\text{n}}^{*}{\mathbf{Z}}_{\text{c}}^{{*}^{\prime}}{\left({\mathbf{Z}}_{\text{c}}^{*}{\mathbf{Z}}_{\text{c}}^{{*}^{\prime}}\right)}^{-1}{\varvec{\upvarepsilon}}+{\varvec{\upxi}}\end{array}\right].$$

Letting $${\mathbf{W}}^{\dagger\mathbf{*}}=\left[\begin{array}{c}{\mathbf{A}}_{12}{\mathbf{A}}_{22}^{-1}{\mathbf{Z}}^{\dagger\mathbf{*}}\\ {\mathbf{Z}}^{\dagger\mathbf{*}}\end{array}\right]$$, $${\mathbf{R}}^{\dagger\mathbf{*}}=\left[\begin{array}{c}{\mathbf{A}}_{12}{\mathbf{A}}_{22}^{-1}\mathbf{R}\\ \mathbf{R}\end{array}\right]$$, and $${\mathbf{Q}}^{\dagger\mathbf{*}}=\left[\begin{array}{c}{\mathbf{A}}_{12}{\mathbf{A}}_{22}^{-1}\mathbf{Q}\\ \mathbf{Q}\end{array}\right]$$, Eq. (33) leads to the following model:

$$\mathbf{y}=\mathbf{X}\mathbf{b}+{\mathbf{W}}^{\dagger\mathbf{*}}\mathbf{a}+{\mathbf{R}}^{\dagger\mathbf{*}}{\varvec{\upvarepsilon}}+\mathbf{T}{\varvec{\upeta}}+{\mathbf{Q}}^{\dagger\mathbf{*}}{\varvec{\upxi}}+\mathbf{e},$$

39 $$\text{Var}\left[\begin{array}{c}\mathbf{a}\\ {\varvec{\upvarepsilon}}\\ {\varvec{\upeta}}\\ {\varvec{\upxi}}\\ \mathbf{e}\end{array}\right]=\left[\begin{array}{ccccc}\left(1-\beta \right)k\mathbf{I}{\upsigma }_{\text{u}}^{2}& \boldsymbol{0}& \boldsymbol{0}& \boldsymbol{0}& \boldsymbol{0}\\ \boldsymbol{0}& \beta {\mathbf{A}}_{\text{cc}}{\upsigma }_{\text{u}}^{2}& \boldsymbol{0}& \boldsymbol{0}& \boldsymbol{0}\\ \boldsymbol{0}& \boldsymbol{0}& {\left({\mathbf{A}}^{11}\right)}^{-1}{\upsigma }_{\text{u}}^{2}& \boldsymbol{0}& \boldsymbol{0}\\ \boldsymbol{0}& \boldsymbol{0}& \boldsymbol{0}& {\mathbf{M}}_{\text{nn}}{\upsigma }_{\text{u}}^{2}& \boldsymbol{0}\\ \boldsymbol{0}& \boldsymbol{0}& \boldsymbol{0}& \boldsymbol{0}& \mathbf{I}{\upsigma }_{\text{e}}^{2}\end{array}\right],$$

with the following MME:

40 $$\left[\begin{array}{ccccc}\mathbf{X}\mathbf{^{\prime}}\mathbf{X}& \mathbf{X}\mathbf{^{\prime}}{\mathbf{W}}^{\dagger\mathbf{*}}& \mathbf{X}\mathbf{^{\prime}}{\mathbf{R}}^{\dagger\mathbf{*}}& {\mathbf{X}}_{1}^{\prime}{\mathbf{W}}_{1}& \mathbf{X}\mathbf{^{\prime}}{\mathbf{Q}}^{\dagger\mathbf{*}}\\ {\mathbf{W}}^{{\dagger\mathbf{*}}^{\mathbf{^{\prime}}}}\mathbf{X}& {\mathbf{W}}^{{\dagger\mathbf{*}}^{\mathbf{^{\prime}}}}{\mathbf{W}}^{\dagger\mathbf{*}}+\mathbf{I}\delta & {\mathbf{W}}^{{\dagger\mathbf{*}}^{\mathbf{^{\prime}}}}{\mathbf{R}}^{\dagger\mathbf{*}}& {\mathbf{W}}^{{\dagger\mathbf{*}}^{\mathbf{^{\prime}}}}\mathbf{T}& {\mathbf{W}}^{{\dagger\mathbf{*}}^{\mathbf{^{\prime}}}}{\mathbf{Q}}^{\dagger\mathbf{*}}\\ {\mathbf{R}}^{{\dagger\mathbf{*}}^{\mathbf{^{\prime}}}}& {\mathbf{R}}^{{\dagger\mathbf{*}}^{\mathbf{^{\prime}}}}& {\mathbf{R}}^{{\dagger\mathbf{*}}^{\mathbf{^{\prime}}}}{\mathbf{R}}^{\dagger\mathbf{*}}+{\mathbf{A}}_{\text{cc}}^{-1}\zeta & {\mathbf{R}}^{{\dagger\mathbf{*}}^{\mathbf{^{\prime}}}}\mathbf{T}& {\mathbf{R}}^{{\dagger\mathbf{*}}^{\mathbf{^{\prime}}}}{\mathbf{Q}}^{\dagger\mathbf{*}}\\ {\mathbf{W}}_{1}^{\prime}{\mathbf{X}}_{1}& {\mathbf{T}}^{\mathbf{^{\prime}}}{\mathbf{W}}^{\dagger\mathbf{*}}& {\mathbf{T}}^{\mathbf{^{\prime}}}{\mathbf{R}}^{\dagger\mathbf{*}}& {\mathbf{W}}_{1}^{\prime}{\mathbf{W}}_{1}+{\mathbf{A}}^{11}\alpha & {\mathbf{T}}^{\mathbf{^{\prime}}}{\mathbf{Q}}^{\dagger\mathbf{*}}\\ {\mathbf{Q}}^{{\dagger\mathbf{*}}^{\mathbf{^{\prime}}}}\mathbf{X}& {\mathbf{Q}}^{{\dagger\mathbf{*}}^{\mathbf{^{\prime}}}}{\mathbf{W}}^{\dagger\mathbf{*}}& {\mathbf{Q}}^{{\dagger\mathbf{*}}^{\mathbf{^{\prime}}}}{\mathbf{R}}^{\dagger\mathbf{*}}& {\mathbf{Q}}^{{\dagger\mathbf{*}}^{\mathbf{^{\prime}}}}\mathbf{T}& {\mathbf{Q}}^{{\dagger\mathbf{*}}^{\mathbf{^{\prime}}}}{\mathbf{Q}}^{\dagger\mathbf{*}}+{\mathbf{M}}_{\text{nn}}^{-1}\alpha \end{array}\right]\left[\begin{array}{c}\widehat{\mathbf{b}}\\ \widehat{\mathbf{a}}\\ \widehat{{\varvec{\upvarepsilon}}}\\ \widehat{{\varvec{\upeta}}}\\ \widehat{{\varvec{\upxi}}}\end{array}\right]=\left[\begin{array}{c}\mathbf{X}\mathbf{^{\prime}}\mathbf{y}\\ {\mathbf{W}}^{{\dagger\mathbf{*}}^{\mathbf{^{\prime}}}}\mathbf{y}\\ {\mathbf{R}}^{{\dagger\mathbf{*}}^{\mathbf{^{\prime}}}}\mathbf{y}\\ {\mathbf{T}}^{\mathbf{^{\prime}}}\mathbf{y}\\ {\mathbf{Q}}^{{\dagger\mathbf{*}}^{\mathbf{^{\prime}}}}\mathbf{y}\end{array}\right]$$

### Computational complexity of the APY-based models

In terms of pre-processing steps, i.e., preparation of the required matrices to set up the MME, the APY-GBLUP model has a cubic cost in the number of core animals, and a linear cost in both the number of markers and the number of non-core animals. In contrast, the APY-SNP-BLUP model has a quadratic cost in both the number of core animals and the number of markers, and a linear cost in the number of non-core animals. Per iteration, the computational cost of APY-GBLUP is quadratic in the number of core and linear in the number of non-core animals, while APY-SNP-BLUP is quadratic in the number of markers and linear in the number of non-core animals. Thus, as long as the number of core animals is smaller than the number of markers, APY-GBLUP will be computationally less demanding than APY-SNP-BLUP. If a residual polygenic effect is assumed, the choice of the core animals plays a major role in computational efficiency because of the computation of ${\mathbf{A}}_{\text{cc}}^{-1}$. For example, if unrelated core animals are chosen, then computations that involve ${\mathbf{A}}_{\text{cc}}^{-1}$ are trivial. Regardless, the APY-SNP-BLUP model may not be of great practical interest, as it can be replaced by either GBLUP or SNP-BLUP. However, the APY-SNP-BLUP model is useful to derive analytical properties of the APY algorithm.

Under single-step models, computational requirements for genetic evaluation of genotyped animals depend on how ${\mathbf{A}}_{22}^{-1}$ is included in the MME for both breeding value and marker-based models. That is, ${\mathbf{A}}_{22}^{-1}$ is included either as the product of ${\mathbf{A}}_{22}^{-1}$ times a vector for breeding value models [26] or as the solution of the sparse system ${\mathbf{A}}^{11}{\widehat{\mathbf{Z}}}^{\dagger}=-{\mathbf{A}}^{12}{\mathbf{Z}}^{\dagger}$ for marker based models [25], where ${\widehat{\mathbf{Z}}}^{\dagger}$ is the matrix of imputed genotypes.

## Discussion

In this study, we derived a marker effects model that is equivalent to the APY-GBLUP model. On the one hand, when the number of core animals is equal to the rank of the genotype matrix, ${\mathbf{G}}_{\text{APY}}$ is singular, as noted by [12]. This can be interpreted as core animals covering all the genetic variation in the population that is captured by the genotyped markers. In that case, APY-GBLUP is equivalent to a regular GBLUP or SNP-BLUP, which has been analytically proven in this study (for further information, a numerical illustration is provided in Additional file 1). On the other hand, when the number of core animals is lower than the rank of the genotype matrix, ${\mathbf{G}}_{\text{APY}}$ is non-singular and a marker effects model, named APY-SNP-BLUP, can be constructed that is equivalent to APY-GBLUP. The APY-SNP-BLUP differs from the regular SNP-BLUP model in the following manner: (i) it has a reduction in the row and column spaces of $\mathbf{Z}$ (from its replacement with ${\mathbf{Z}}^{\dagger}$), and (ii) it has an additional error term $\left({\varvec{\upxi}}\right)$ for non-core animals. The former indicates that the number of possible genotypes and haplotypes that can be formed by a linear combination of the rows or columns of the genotype matrix is reduced. The degree of reduction of both spaces is controlled by the number of core animals. With a fixed number of core animals, selecting different sets of core animals is equivalent to selecting different subspaces of the row and column spaces of $\mathbf{Z}$. When the number of core animals is such that a large portion of the spectrum of $\mathbf{G}$ (or the set of singular values of $\mathbf{Z}$) is covered, those sub-spaces that correspond to different sets of core animals will increasingly overlap, which means that their intersection will not be equal to the null vector space. In that case, many genotypes in the population can be generated as linear combinations of the rows of ${\mathbf{Z}}_{\text{c}}$. If a certain genotype, say ${\mathbf{z}}_{i}$, cannot be formed, the distance between ${\mathbf{z}}_{i}$ and its projection to the row space of ${\mathbf{Z}}_{\text{c}}\left(d\left({\mathbf{z}}_{i}-\varvec{\mathcal{P}}{\mathbf{z}}_{i}\right)\right)$ is in general expected to be small. The particular choice of core animals is not important, as long as it covers the spectrum of $\mathbf{G}$. Although the heuristics of how core animals should be chosen needs to be refined, a wide random choice appears to be adequate [10, 13, 27, 28]. However, as the population evolves and new haplotype combinations are created, the chosen core may become less representative, although this is expected to be a slow process. Methods to choose and update a set of core animals that spans most variability in $\mathbf{G},$ e.g., [29], is an open area of research.

The large changes in estimated breeding values for some non-core animals when the core animals are changed, while keeping its number constant, as reported by [30], can be explained by a large distance between the projected and the real genotype of those individuals. This is the case, for instance, when a mislabeled animal is evaluated within one breed but actually belongs to another breed.

Note also that core animals do not need to be individuals with, a priori, high reliability. Marker effects are back-solved from core animals, but these animals gather information from the entire population through the genomic relationships. Thus, the accuracy of the estimated breeding values of these core animals will be very high. For instance, in dairy cattle, core animals could be based on cows. If these cows have a strong relationship with the whole population, they are very accurately estimated, and so will be the SNP effects and indirect predictions.

We also derived the distribution of breeding values conditional on the marker effects and vice versa when using APY. For the first case, breeding values of the non-core animals are only partially explained by the marker effects because of the error term ${\varvec{\upxi}}$ in Eq. (10). For the second case, we showed that the BLUP of the marker effects conditional on the breeding values of animals, only requires the matrices and estimated breeding values corresponding to the core animals—this result was not known before. Although non-core animals do not appear in the explicit calculation of the marker effects, their information is used to estimate breeding values of the core animals. When Eq. (21) is not used to obtain estimates for the marker effects from an APY-GBLUP model, those estimates will not have minimum variance.

In genetic evaluations, indirect predictions for animals without own records or progeny with records can be calculated from estimates of marker effects that are obtained by back-solving from the estimated breeding values [20]. If an APY-(ss)GBLUP model is used for genetic evaluations then the proper way to calculate indirect predictions is based on the distribution in Eq. (18). For core animals, the formula to calculate indirect predictions is equal to the Eq. (10) in [20]. However, for genetic evaluation, animals for which indirect predictions are calculated are, by definition, non-core animals. Therefore, in that case, their indirect predictions must be obtained from Eq. (23).

Two measures of uncertainty associated with indirect predictions were derived. The first measure of uncertainty, Eq. (24), quantifies how different the indirect prediction would be from that calculated with a regular GBLUP or SNP-BLUP model. If the number of core animals is large enough, this measure will be close to 1. A value of 1 indicates that the individual is in the space of genetic variation described by the core animals and indirect predictions based on APY and SNP-BLUP are identical. The second measure of uncertainty, based on Eq. (26), is a classical reliability of estimated breeding value and is a function of the prediction error variance of the indirect prediction in Eq. (25). This reliability can be expressed in terms of prediction error variances of marker effects or in terms of prediction error variances of core animals. The latter results in higher computational efficiency because the number of core animals is smaller than the number of markers.

In the same way that an equivalent SNP-BLUP model exists for the APY-BLUP model, we showed that when genotyped and non-genotyped animals are combined in the evaluation using a single-step approach, there is an APY-ssSNP-BLUP model that is equivalent to the APY-ssGBLUP model. In the APY-ssGBLUP model, breeding values are jointly estimated for non-genotyped, core, and non-core animals, while the APY-ssSNP-BLUP model estimates SNP effects based on the core animals, an error term for non-core animals, and the genotype imputation error for non-genotyped animals. Estimates of breeding values for all the animals can then be obtained by a linear combination of the corresponding design matrices and the vector of solutions. As in Fernando et al. [31], estimated breeding values for non-genotyped animals can be obtained directly to improve computational efficiency. Adding the polygenic effect does not change the MME for APY-ssGBLUP but adds an extra term ($\widehat{{\varvec{\upvarepsilon}}}$) to the MME for APY-ssSNP-BLUP. In that case, the number of unknowns is equal to that in the model presented by [22]. Therefore, APY-ssSNP-BLUP is more complex and involves more convoluted matrix multiplications. Whenever the MME are augmented, there is a question on the feasibility and convergence of the model with real datasets. Vandenplas et al. [5] proposed a second-level preconditioner to ease convergence problems in ssSNP-BLUP, and Vandenplas et al. [6] presented a different termination criterion to determine convergence of such models. Although APY-ssSNP-BLUP is more flexible regarding the use of different priors for marker effects [25], its convergence will need to be monitored carefully.

## Conclusions

The APY-GBLUP model is equivalent to a family of marker effect models that are here described as APY-SNP-BLUP. We show that when the choice of core animals covers the rank of the genotype matrix, which is generally equal to the number of markers, APY-GBLUP is equivalent to a conventional SNP-BLUP model and is, therefore, just as accurate. If the choice of core animals does not cover the spectrum of the genomic relationship matrix, the genotypes for the non-core animals are imputed as a linear combination of the genotypes of the core animals. Marker effect estimates for the APY-SNP-BLUP model can then be calculated by a linear transformation of the estimated breeding values of the core animals. Thus, all the matrices involved have a size equal to the number of core animals. Indirect predictions for non-core animals with APY can be calculated from estimated marker effects from APY-SNP-BLUP, or from estimated breeding values for core animals, without a need to consider non-core animals, which simplifies the calculations. The reliability of indirect predictions is solely a function of the prediction error variance of the estimated breeding values of core animals. Therefore, choosing core animals that cover correctly the spectra of the genotype matrix will give indirect predictions with high reliability. Both the APY-GBLUP and the APY-SNP-BLUP models can fit a residual polygenic effect, and when the APY-SNP-BLUP is used, only residual polygenic effects for core animals are fitted.

## Supplementary Information

**Additional file 1. **Weighted marker effects in APY-SNP-BLUP and numerical illustration of the equivalence between APY-GBLUP and APY-SNP-BLUP with and without a residual polygenic effect.

## Data Availability

Not applicable.
