# Supplementary material for: On the equivalence between marker effect models and breeding value models and direct genomic values with the Algorithm for Proven and Young
Source: Genet Sel Evol. 2022 Jul 16;54:52. doi: 10.1186/s12711-022-00741-7 (PMC9288049; doi:10.1186/s12711-022-00741-7)
Supplement: Supplementary file 1 — Additional file 1. Weighted marker effects in APY-SNP-BLUP and numerical illustration of the equivalence between APY-GBLUP and APY-SNP-BLUP with and without a residual polygenic effect. [file 12711_2022_741_MOESM1_ESM.docx]

***Marker effects in APY-SNP-BLUP with weights***

The SNP effects $\left( \mathbf{a} \right)$ can be weighted by calculating the genomic relationship matrix as $\mathbf{G}=\mathbf{ZDZ}'$, where $\mathbf{D}$ is a diagonal matrix. In such a case, the variance of $\mathbf{a}$ in Eq. (12) is replaced by $\mathbf{D}\sigma_{u}^{2}$, and $\mathcal{P}\mathbf{=}\mathbf{Z}_{c}^{\mathbf{'}}\left( \mathbf{Z}_{c}\mathbf{Z}_{c}^{\mathbf{'}} \right)^{-1}\mathbf{Z}_{c}$ by $\mathcal{P}\mathbf{=}\mathbf{Z}_{c}^{\mathbf{'}}\left( \mathbf{Z}_{c}\mathbf{D}\mathbf{Z}_{c}^{\mathbf{'}} \right)^{-1}\mathbf{Z}_{c}\mathbf{D}$ (i.e., the perpendicular projector operator is replaced by an oblique projector operator). Then, Eq. (17) turns into $\mathrm{Var}\left[ \begin{matrix} \mathbf{u} \\ \mathbf{a} \end{matrix} \right]=\left[ \begin{matrix} \mathbf{Z}^{\dagger}\mathbf{D}\mathbf{Z}^{\dagger^{'}}+\mathbf{V} & \mathbf{Z}^{\dagger}\mathbf{D} \\ \mathbf{D}\mathbf{Z}^{\dagger^{'}} & \mathbf{D} \end{matrix} \right]\sigma_{u}^{2}$. Which after algebra leads to $\hat{\mathbf{a}}\mathbf{|}\hat{\mathbf{u}}\mathbf{=D}\mathbf{Z}_{c}^{\mathbf{'}}\mathbf{G}_{\mathrm{cc}}^{-1}{\hat{\mathbf{u}}}_{c}$. Since $\mathbf{D}$ is positive definite, another alternative would be updating $\mathbf{Z}$ as $\mathbf{Z=Z}\mathbf{D}^{1/2}$ and hold the same notation as in Eq. (20) and Eq. (21) but without $k$.

***Relationship between*** $\boldsymbol{rel}_{\boldsymbol{i}}$ ***and*** $\boldsymbol{\rho}_{\boldsymbol{i}}$

From Eq. (26):

| $\mathrm{rel}_{i}\boldsymbol{=}1-\frac{\mathbf{m}_{\mathrm{ii}}\boldsymbol{+}\mathbf{g}_{i,c}\mathbf{G}_{\mathrm{cc}}^{-1}\mathrm{PEV}_{\mathrm{core}}\mathbf{G}_{\mathrm{cc}}^{-1}\mathbf{g}_{c,i}}{\mathbf{g}_{\mathrm{ii}}}=\frac{\mathbf{g}_{\mathrm{ii}}\boldsymbol{-}\mathbf{g}_{\mathrm{ii}}+\mathbf{g}_{i,c}\mathbf{G}_{\mathrm{cc}}^{-1}\mathbf{g}_{c,i}\boldsymbol{-}\mathbf{g}_{i,c}\mathbf{G}_{\mathrm{cc}}^{-1}\mathrm{PEV}_{\mathrm{core}}\mathbf{G}_{\mathrm{cc}}^{-1}\mathbf{g}_{c,i}}{\mathbf{g}_{\mathrm{ii}}}=\frac{\mathbf{g}_{i,c}\mathbf{G}_{\mathrm{cc}}^{-1}\mathbf{g}_{c,i}}{\mathbf{g}_{\mathrm{ii}}}-\frac{\mathbf{g}_{i,c}\mathbf{G}_{\mathrm{cc}}^{-1}\mathrm{PEV}_{\mathrm{core}}\mathbf{G}_{\mathrm{cc}}^{-1}\mathbf{g}_{c,i}}{\mathbf{g}_{\mathrm{ii}}}=\frac{\mathbf{g}_{\mathrm{ii}}-\mathbf{m}_{\mathrm{ii}}}{\mathbf{g}_{\mathrm{ii}}}-\frac{\mathbf{g}_{i,c}\mathbf{G}_{\mathrm{cc}}^{-1}\mathrm{PEV}_{\mathrm{core}}\mathbf{G}_{\mathrm{cc}}^{-1}\mathbf{g}_{c,i}}{\mathbf{g}_{\mathrm{ii}}}=1-\frac{\mathbf{m}_{\mathrm{ii}}}{\mathbf{g}_{\mathrm{ii}}}-\frac{\mathbf{g}_{i,c}\mathbf{G}_{\mathrm{cc}}^{-1}\mathrm{PEV}_{\mathrm{core}}\mathbf{G}_{\mathrm{cc}}^{-1}\mathbf{g}_{c,i}}{\mathbf{g}_{\mathrm{ii}}}=\rho_{i}-\frac{\mathbf{g}_{i,c}\mathbf{G}_{\mathrm{cc}}^{-1}\mathrm{PEV}_{\mathrm{core}}\mathbf{G}_{\mathrm{cc}}^{-1}\mathbf{g}_{c,i}}{\mathbf{g}_{\mathrm{ii}}}$ | (1) |
| --- | --- |

***Numerical example of the equivalence between models***

Numerical examples were constructed based on the pedigree and genotypes for six animals shown in Table 1. All animals had phenotypes composed by the sum of an overall mean (μ = 2.0), breeding values, and a residual; therefore, the only fixed effect in the model was the overall mean. Both random effects were assumed to follow a standard normal distribution, resulting in a variance ratio equal to one. The value of $k$ was assumed to be 0.5. Tables 2-3 and 4-5 show the equivalence between APY-GBLUP and SNP-BLUP. The numerical examples to show the equivalence between models with a residual polygenic effect are in Tables 6 and 7. The proportion of the residual polygenic component was assumed to be equal to 0.05.

**Table 1.** Pedigree and genotypes for four markers (M1 – M4) observed in six individuals.

| Animal | Sire | Dam | Genotypes | | | |
| --- | --- | --- | --- | --- | --- | --- |
|  |  |  | M1 | M2 | M3 | M4 |
| 1 | 0 | 0 | -1 | 0 | 1 | -1 |
| 2 | 0 | 0 | 0 | 1 | 0 | 0 |
| 3 | 0 | 0 | 0 | -1 | -1 | -1 |
| 4 | 1 | 2 | 0 | 0 | 1 | -1 |
| 5 | 1 | 3 | 0 | -1 | 0 | -1 |
| 6 | 4 | 5 | -1 | 0 | 0 | -1 |

**Table 2.** Mixed model equations (MME)^1,2^, right-hand side (RHS), and solutions (SOL) for APY-GBLUP with animals 1 and 2 as core animals.

| MME | | | | | | | RHS | SOL |
| --- | --- | --- | --- | --- | --- | --- | --- | --- |
| 6 | 1 | 1 | 1 | 1 | 1 | 1 | 11.54 | 1.99 |
| 1 | 4.67 | -1 |  | -2 | -1 | -2 | 1.2 | 0.05 |
| 1 | -1 | 7 | 1 |  | 3 |  | 2.79 | 0.62 |
| 1 |  | 1 | 2 |  |  |  | 0.95 | -0.83 |
| 1 | -2 |  |  | 4 |  |  | 3.68 | 0.44 |
| 1 | -1 | 3 |  |  | 4 |  | 0.21 | -0.90 |
| 1 | -2 |  |  |  |  | 4 | 2.71 | 0.20 |

^1^ The model includes an overall mean whose estimate is located in the first position of the solution vector. The rest of the effects represent the animals from Table 1, ordered by identification.

^2^ A variance ratio equal to the unity was used for the MME.

**Table 3.** Mixed model equations (MME)^1,2,3^, right-hand side (RHS), vector of solutions (SOL), and estimated breeding values (EBV) for APY-SNP-BLUP with animals 1 and 2 as core animals.

| MME | | | | | | | | | RHS | SOL |
| --- | --- | --- | --- | --- | --- | --- | --- | --- | --- | --- |
| 6 | -2.67 | -1 | 2.67 | -2.67 | 1 | 1 | 1 | 1 | 11.54 | 1.99 |
| -2.67 | 4 | 0.33 | -2 | 2 |  | -0.67 | -0.33 | -0.67 | -5.52 | -0.01 |
| -1 | 0.33 | 5 | -0.33 | 0.33 | -1 |  | -1 |  | 1.63 | 0.62 |
| 2.67 | -2 | -0.33 | 4 | -2 |  | 0.67 | 0.33 | 0.67 | 5.52 | 0.01 |
| -2.67 | 2 | 0.33 | -2 | 4 |  | -0.67 | -0.33 | -0.67 | -5.52 | -0.01 |
| 1 |  | -1 |  |  | 2 |  |  |  | 0.95 | -0.20 |
| 1 | -0.67 |  | 0.67 | -0.67 |  | 4 |  |  | 3.68 | 0.41 |
| 1 | -0.33 | -1 | 0.33 | -0.33 |  |  | 4 |  | 0.21 | -0.29 |
| 1 | -0.67 |  | 0.67 | -0.67 |  |  |  | 4 | 2.71 | 0.17 |
| EBV | | | | | | | | | | |
|  |  | 1 | 2 | 3 | 4 | 5 | 6 |  |  |  |
|  |  | 0.05 | 0.62 | -0.83 | 0.44 | -0.90 | 0.20 |  |  |  |

^1^ The model includes an overall mean whose estimate is located in the first position of the solution vector. The effects 2 to 4 represent the marker effects, whereas the remainder represents the errors for non-core animals.

^2^ A variance ratio equal to the unity was used for the MME.

^3^ *k* was equal to 0.5.

**Table 4.** Mixed model equations (MME)^1,2,3^, right-hand side (RHS), and solutions (SOL) for APY-GBLUP with animals 1 to 4 as core animals.

| MME | | | | | | | RHS | SOL |
| --- | --- | --- | --- | --- | --- | --- | --- | --- |
| 6 | 1 | 1 | 1 | 1 | 1 | 1 | 11.54 | 2.05 |
| 4 | 2.5 |  |  | 1 | 0.5 | 1 | 8.29 | 0.13 |
| -0.5 |  | 1.5 | -0.5 |  | -0.5 |  | 0.81 | 0.66 |
| 2.5 |  | -0.5 | 2.5 |  | 1 | 0.5 | 1.59 | -0.97 |
| 3 | 1 |  |  | 2 | 0.5 | 0.5 | 6.34 | 0.23 |
| 3 | 0.5 | -0.5 | 1 | 0.5 | 2 | 0.5 | 3.56 | -0.70 |
| 3.5 | 1 |  | 0.5 | 0.5 | 0.5 | 2 | 6.33 | -0.13 |

^1^ The model includes an overall mean whose estimate is located in the first position of the solution vector. The rest of the effects represent the animals from Table 1, ordered by identification.

^2^ A variance ratio equal to the unity was used for the MME.

^3^ Non-symmetric MME were used due to the singularity of the covariance matrix for the random effects.

**Table 5.** Mixed model equations (MME)^1,2,3,4^, right-hand side (RHS), vector of solutions (SOL), and estimated breeding values (EBV) for APY-SNP-BLUP with animals 1 to 4 as core animals.

| MME | | | | | RHS | SOL |
| --- | --- | --- | --- | --- | --- | --- |
| 6 | -2 | -1 | 1 | -5 | 11.54 | 2.05 |
| -2 | 4 |  | -1 | 2 | -3.91 | 0.09 |
| -1 |  | 5 | 1 | 2 | 1.63 | 0.66 |
| 1 | -1 | 1 | 5 | -1 | 3.93 | 0.26 |
| -5 | 2 | 2 | -1 | 7 | -8.75 | 0.03 |
| Estimated breeding values | | | | | | |
| 1 | 2 | 3 | 4 | 5 | 6 |  |
| 0.13 | 0.66 | -0.97 | 0.23 | -0.70 | -0.13 |  |

^1^ The model includes an overall mean whose estimate is located in the first position of the solution vector. The effects 2 to 4 represent the marker effects.

^2^ A variance ratio equal to the unity was used for the MME.

^3^ *k* was equal to 0.5.

^4^ The error term of non-core animals is null because the number of core animals equals to the rank of the genotyped matrix, as shown in (14) to (16).

**Table 6.** Mixed model equations (MME)^1,2^, right-hand side (RHS), and solutions (SOL) for APY-GBLUP with a residual polygenic effect^3^ with animals 1 and 2 as core animals.

| MME | | | | | | | RHS | SOL |
| --- | --- | --- | --- | --- | --- | --- | --- | --- |
| 6 | 1 | 1 | 1 | 1 | 1 | 1 | 11.54 | 1.97 |
| 1 | 4.40 | -0.63 |  | -1.86 | -0.84 | -1.82 | 1.2 | 0.04 |
| 1 | -0.63 | 5.73 | 0.86 | -0.13 | 2.25 | -0.06 | 2.79 | 0.63 |
| 1 |  | 0.86 | 1.95 |  |  |  | 0.95 | -0.80 |
| 1 | -1.86 | -0.13 |  | 3.82 |  |  | 3.68 | 0.48 |
| 1 | -0.84 | 2.25 |  |  | 3.49 |  | 0.21 | -0.90 |
| 1 | -1.82 | -0.06 |  |  |  | 3.76 | 2.71 | 0.22 |

^1^ The model includes an overall mean whose estimate is located in the first position of the solution vector. The rest of the effects represent the animals from Table 1, ordered by identification.

^2^ A variance ratio equal to the unity was used for the MME.

^3^ The proportion of residual polygenic effect was assumed equal to 0.05.

**Table 7.** Mixed model equations (MME)^1,2,3^, right-hand side (RHS), vector of solutions (SOL), and estimated breeding values (EBV) for APY-SNP-BLUP with a random polygenic effect^4^ with animals 1 and 2 as core animals.

| MME | | | | | | | | | | | RHS | SOL |
| --- | --- | --- | --- | --- | --- | --- | --- | --- | --- | --- | --- | --- |
| 6 | -2.67 | -0.73 | 2.67 | -2.67 | 2.67 | -0.73 | 1 | 1 | 1 | 1 | 11.54 | 1.97 |
| -2.67 | 4.09 | 0.25 | -1.98 | 1.98 | -1.98 | 0.25 |  | -0.66 | -0.33 | -0.66 | -5.49 | -0.01 |
| -0.73 | 0.25 | 4.74 | -0.25 | 0.25 | -0.25 | 2.64 | -0.90 | 0.04 | -0.90 | 0.02 | 1.98 | 0.57 |
| 2.66 | -1.98 | -0.25 | 4.09 | -1.98 | 1.98 | -0.25 |  | 0.66 | 0.33 | 0.66 | 5.49 | 0.01 |
| -2.66 | 1.98 | 0.25 | -1.98 | 4.09 | -1.98 | 0.25 |  | -0.66 | -0.33 | -0.66 | -5.49 | -0.01 |
| 2.66 | -1.98 | -0.25 | 1.98 | -1.98 | 21.98 | -0.25 |  | 0.66 | 0.33 | 0.66 | 5.49 | 0.001 |
| -0.73 | 0.25 | 2.64 | -0.25 | 0.25 | -0.25 | 22.64 | -0.90 | 0.04 | -0.90 | 0.02 | 1.98 | 0.06 |
| 1 |  | -0.90 |  |  |  | -0.90 | 1.95 |  |  |  | 0.95 | -0.22 |
| 1 | -0.66 | 0.04 | 0.66 | -0.66 | 0.66 | 0.04 |  | 3.82 |  |  | 3.68 | 0.43 |
| 1 | -0.33 | -0.90 | 0.33 | -0.33 | 0.33 | -0.90 |  |  | 3.49 |  | 0.21 | -0.34 |
| 1 | -0.66 | 0.02 | 0.66 | -0.66 | 0.66 | 0.02 |  |  |  | 3.76 | 2.71 | 0.18 |
| EBV | | | | | | | | | | | | |
|  |  |  | 1 | 2 | 3 | 4 | 5 | 6 |  |  |  |  |
|  |  |  | 0.04 | 0.63 | -0.80 | 0.48 | -0.90 | 0.22 |  |  |  |  |

^1^ The model includes an overall mean whose estimate is located in the first position of the solution vector. The effects 2 to 4 represent the marker effects, effects 5 and 6 represent the residual polygenic effects for the core animals, and the remainder of the effects represents the errors for non-core animals.

^2^ A variance ratio equal to the unity was used for the MME.

^3^ *k* was equal to 0.5.

^3^ The proportion of residual polygenic effect was assumed equal to 0.05.
